# Development and Implementation of a Hospital-Wide Multidisciplinary Team Care Model in a Tertiary General Hospital in China: A Project Report

**DOI:** 10.3390/healthcare14213728

**Published:** 2026-11-03

**Authors:** Gongming Zhao, Xinyu Tao, Chen Qu, Ling Zhao

**Affiliations:** 1 Department of Clinical Medicine, Nanjing Medical University, Nanjing 211166, China; zgm788004@163.com; 2 Department of Geriatrics, The Second Affiliated Hospital of Nanjing Medical University, Nanjing 210011, China; 18895600220@163.com; 3 Department of Organization and Personnel, The Second Affiliated Hospital of Nanjing Medical University, Nanjing 210011, China

**Keywords:** multidisciplinary team, integrated care, interprofessional collaboration, hospital management, healthcare organization, implementation, tertiary hospital, China

## Abstract

Background: Patients with multimorbidity, multisystem disease, rare conditions, or competing treatment priorities frequently require coordination beyond the scope of a single specialty. This Project Report describes the development and implementation of a hospital-wide multidisciplinary team (MDT) care model at a tertiary general hospital in China. Methods: The report synthesizes institutional documents and routine program records describing MDT governance, workflow, digital support, operational activity, and implementation experience. Quantitative indicators for 2021–2025 were summarized descriptively. No comparator group was included, and no causal effectiveness analysis or inferential statistical testing was performed. Results: Initiated with a geriatric medicine MDT in 2015, the program subsequently developed into a hospital-wide framework comprising seven clinical-domain-based MDT centers by 2023. The number of participating departments increased from 4 in 2021 to 23 in 2025, while the annual number of patients receiving MDT care increased overall from 32 to 223. Virtual MDT predominated during 2021–2022, whereas on-site MDT became the predominant mode from 2023 onward. MDT sessions involving trainees increased from 15 in 2021 to 119 in 2025. The program incorporated standardized case selection, structured multidisciplinary review, documented consensus planning, follow-up and repeat review, and digital support for information sharing and remote participation. Implementation experience highlighted practical challenges related to expert coordination, multidisciplinary consensus, information completeness, follow-up, clinician participation, and cross-campus coordination. Conclusions: The experience indicates that hospital-wide MDT care functions as an institutional coordination system rather than an isolated case-conference mechanism. Implementation of such a model may depend on governance, standardized workflows, reliable information exchange, administrative coordination, clinician participation, and closed-loop follow-up. Prospective evaluation is required to determine effects on clinical, process, patient-reported, and economic outcomes.

## 1. Introduction

The organization of modern hospital care has been increasingly shaped by specialization. Although specialization enhances technical expertise, it may also fragment decision-making when patients present with multimorbidity, multisystem disease, functional vulnerability, or competing therapeutic priorities. Multimorbidity is common in adult populations and particularly prevalent among older people, while health systems have traditionally been organized around individual diseases rather than combinations of conditions [1]. This mismatch underscores the need for more integrated models of care. The World Health Organization’s framework on integrated people-centered health services emphasizes care that responds to individual needs across the life course and is coordinated across the continuum of care [2]. Consistent with this approach, integrated-care theory stresses connectivity, alignment, and collaboration across organizational, service-delivery, and clinical levels [3].

Multidisciplinary team (MDT) care and other interprofessional care models represent organizational responses to this challenge by bringing together professionals with complementary expertise to support communication and joint care planning. However, assembling professionals from different disciplines does not itself ensure effective collaboration; performance depends on how such collaboration is organized and implemented. A meta-review identified facilitators and barriers at organizational, team, and individual levels [4]. A Cochrane review likewise found mixed effects and low- to very-low-certainty evidence for practice-based interprofessional collaboration interventions, indicating that such approaches should not be assumed to improve clinical or service outcomes in every setting [5]. This uncertainty is also evident in oncology, where MDT meetings can influence diagnostic assessment and management decisions, but evidence for consistent effects on survival and other clinical outcomes remains limited or heterogeneous [6,7].

These findings shift attention from the mere presence of an MDT to the conditions under which multidisciplinary care is delivered. The implementation question is therefore not simply whether an MDT meeting is held, but how an institution establishes governance, selects appropriate cases, ensures relevant information is available, structures discussion, documents decisions, coordinates implementation, and maintains follow-up. Evidence from China suggests that these organizational and process dimensions remain insufficiently understood. A systematic review of teamwork in Chinese hospitals found that more than 70% of included studies were conducted in tertiary hospitals and nearly all in urban settings, while evidence linking teamwork inputs, processes, and outcomes remained limited and often inconclusive [8]. A subsequent qualitative study in county-level hospitals identified leadership and task design as major influences on team functioning and described interventions, including simulation training and continuous improvement of team processes [9].

Against this background, The Second Affiliated Hospital of Nanjing Medical University has developed an MDT program since 2015, beginning in geriatric medicine and subsequently expanding across multiple clinical domains. The present report describes how MDT services across oncological and non-oncological domains are organized within a common hospital-level coordination framework, including governance responsibilities, standardized workflow, information support, follow-up arrangements, and multi-year operational reporting. Rather than attempting to establish comparative clinical effectiveness, the aim is to document implementation practices and organizational lessons that may be relevant to other tertiary general hospitals coordinating care for complex patients.

## 2. Materials and Methods

### 2.1. Design and Reporting Approach

This article presents a descriptive Project Report of the implementation of a hospital-wide MDT care model. The institutional program, rather than an experimental or observational patient cohort, constitutes the primary unit of reporting. Accordingly, the report focuses on the development of the program, the organizational and governance structures established to support implementation, the standardized workflow through which MDT care is delivered, and the operational mechanisms used to sustain the program.

Quantitative program indicators were analyzed descriptively to characterize program scale, service delivery, organizational participation, and educational involvement rather than to evaluate treatment effectiveness or support causal inference. No comparator group was included, and no inferential statistical analyses were performed. Counts and percentages were reported where appropriate. Reporting was informed by selected elements of the Standards for Quality Improvement Reporting Excellence (SQUIRE) 2.0, particularly the description of the problem and aims, institutional context and MDT arrangements, operational definitions and descriptive analysis, program development, and interpretation and limitations [10]. These elements were used to structure a descriptive institutional account, rather than to claim full adherence to all SQUIRE items. The report did not evaluate the impact of the MDT model or attribute observed changes to its implementation. Clinical and economic outcomes and long-term sustainability remain priorities for future evaluation.

### 2.2. Institutional Setting and Project Scope

The project was developed at The Second Affiliated Hospital of Nanjing Medical University, a tertiary general hospital integrating clinical care, teaching, and research. The hospital has approximately 2500 authorized beds and more than 3000 employees across multiple medical campuses. The hospital-wide MDT program was designed to coordinate care for patients whose diagnostic or therapeutic needs extend across conventional specialty boundaries.

The institutional model is organized around formally established clinical-domain-based MDT centers supported by participating departments across the hospital. Depending on the clinical problem, individual cases may involve specialists from multiple departments, and the same department may contribute to more than one MDT center. The program is designed to support multidisciplinary coordination from diagnostic assessment and treatment planning through implementation and follow-up, including repeat multidisciplinary review or rehabilitation when clinically indicated.

### 2.3. Data Sources, Operational Definitions, and Descriptive Analysis

This report draws on institutional documents and routine program records related to the development and operation of the hospital-wide MDT program, including MDT management measures, standardized application and consultation workflows, documentation of the hospital information system and MDT management platform, annual administrative records, and education and training records.

Quantitative operational indicators were summarized for 2021–2025 because sufficiently complete and comparable annual operational data could not be reliably reconstructed for the earlier implementation period (2015–2020). The same operational definitions and counting rules were applied throughout 2021–2025. Patients receiving MDT care were de-duplicated within each calendar year, while the same patient could be counted again in a subsequent year. On-site and virtual MDT were classified according to the mode of the patient’s first MDT encounter in that calendar year. Virtual MDT referred to patient-level MDT care delivered through the hospital’s online consultation system; remote participation by external specialists was treated as a separate digital function. MDT centers were defined as formally established clinical-domain-based organizational units, and participating departments were counted as unique departments at the hospital level within each reporting year.

An MDT session was classified as involving trainees when at least one trainee attended. Trainees included residents in standardized training programs, visiting trainees, and interns who were not regular hospital staff, and were counted as unique individuals within each calendar year. All quantitative indicators were summarized descriptively using counts and, where appropriate, percentages and were used to characterize program reach, service delivery, organizational participation, and educational involvement rather than clinical or educational effectiveness.

### 2.4. Synthesis of Implementation Experience

Implementation challenges, institutional responses, and practical implications were summarized descriptively by the author team using institutional policies, routine MDT program records, internal meeting and discussion materials, and relevant hospital reports obtained from administrative departments and clinical MDTs. The materials primarily covered 2021–2025, supplemented by earlier institutional documents describing program development. These materials were considered based on their relevance to MDT organization, workflows, and implementation. Operational issues identified in these materials were organized for reporting into major domains, including expert scheduling and participation, cross-specialty disagreement, case selection and application quality, completeness and accessibility of clinical information, implementation and follow-up of MDT recommendations, patient and family communication, sustained clinician participation, and cross-campus or remote coordination.

Corresponding institutional responses were identified from established management procedures, workflow arrangements, digital support mechanisms, and routine operational practices. Practical implications were synthesized by the authors from these documented implementation experiences to summarize organizational features and operational considerations relevant to the hospital-wide MDT program. This synthesis was descriptive and was not conducted as a formal qualitative study; no interview-based qualitative analysis or formal thematic coding was performed.

### 2.5. Ethical and Data-Governance Considerations

Only aggregated operational data generated through routine management of the hospital-wide MDT program were used for manuscript preparation and analysis. Patient identification and de-duplication, where required for routine annual reporting, were performed within the institutional data-management process before aggregation. No individually identifiable patient information or patient-level clinical outcomes were included in the analytic data used for this report.

The report involved no additional patient contact, research-specific examinations or interventions, or individual-level analysis of treatment effectiveness. The Ethics Committee of The Second Affiliated Hospital of Nanjing Medical University agreed that this report was eligible for exemption from ethical review. Access to the underlying institutional records was restricted to authorized hospital personnel, and only aggregated data were used for reporting.

## 3. Results

### 3.1. Evolution from Specialty-Based MDTs to a Hospital-Wide Program

**Figure 1:**
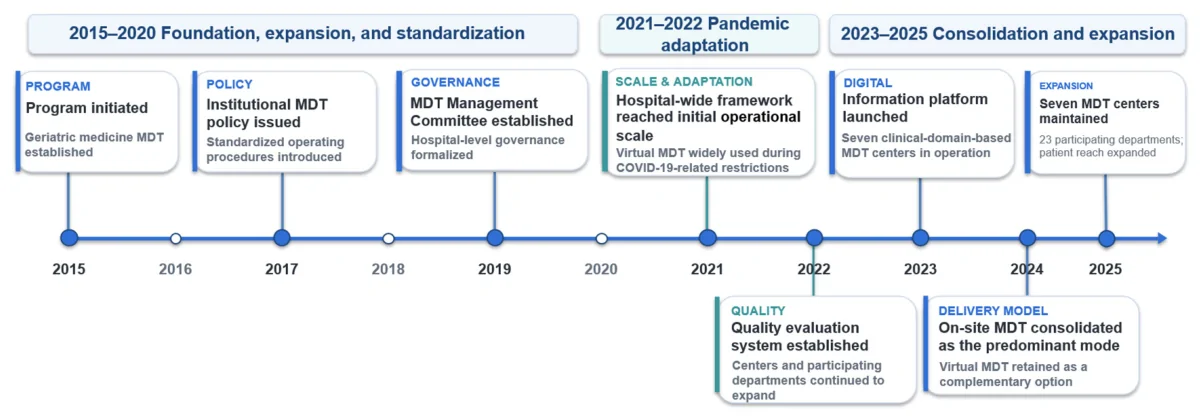
Development of the hospital-wide MDT program, 2015–2025. The timeline summarizes key institutional milestones and broad phases of the MDT program development at The Second Affiliated Hospital of Nanjing Medical University, including program initiation, establishment of institutional governance and quality-management mechanisms, adaptation of service delivery during the COVID-19 pandemic, implementation of digital support, and subsequent consolidation and expansion of the hospital-wide framework.

The hospital-wide MDT program originated from a geriatric medicine MDT initiated in 2015 and subsequently expanded across additional clinical domains. Over time, these specialty-based activities were progressively incorporated into a more standardized hospital-wide framework. By 2023, the program had expanded to seven clinical-domain-based MDT centers, while the number of participating departments continued to increase thereafter. Figure 1 summarizes the major stages in the development of the hospital-wide MDT program from 2015 to 2025.

### 3.2. Organizational Structure and Accountability

The hospital-wide MDT program is organized around seven clinical-domain-based MDT centers. Each center designates a lead specialty, whose academic leader serves as the center director and provides clinical leadership for multidisciplinary case management. The director coordinates the expertise required for individual cases, while participating departments contribute specialists according to clinical need. Individual departments may participate in more than one MDT center, reflecting the cross-cutting nature of the hospital-wide model.

At the hospital level, the MDT Management Committee provides overall governance and is supported by three functional components. The MDT Center Office coordinates case registration, meeting arrangements, and follow-up; the Quality Management Group supports quality monitoring and program improvement activities; and the Information Support Group provides digital infrastructure, information sharing, and support for virtual MDT services. MDT participation is recognized as part of clinicians’ routine workload and professional contributions.

**Figure 2:**
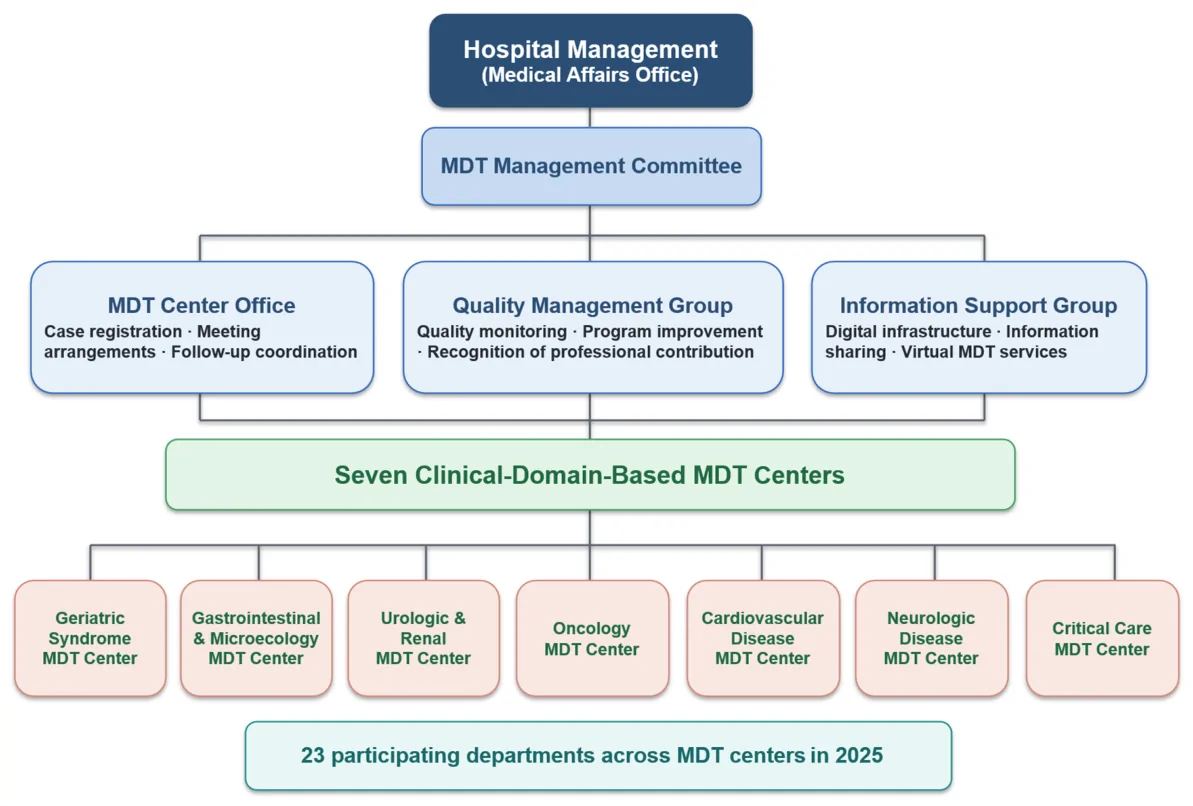
Organizational and governance structure of the hospital-wide MDT program, showing hospital-level coordination, supporting functions, seven clinical-domain-based MDT centers, and participating departments.

This structure combines clinical leadership at the MDT-center level with institutional governance and administrative support at the hospital level. Figure 2 presents the governance framework and the seven MDT centers included in the hospital-wide program. The composition of MDT centers and participating departments was verified through institutional records.

The figure summarizes the hospital-level governance structure, functional support components, and the relationships between the seven clinical-domain-based MDT centers and participating departments.

### 3.3. Case Selection and Standardized MDT Workflow

**Figure 3:**
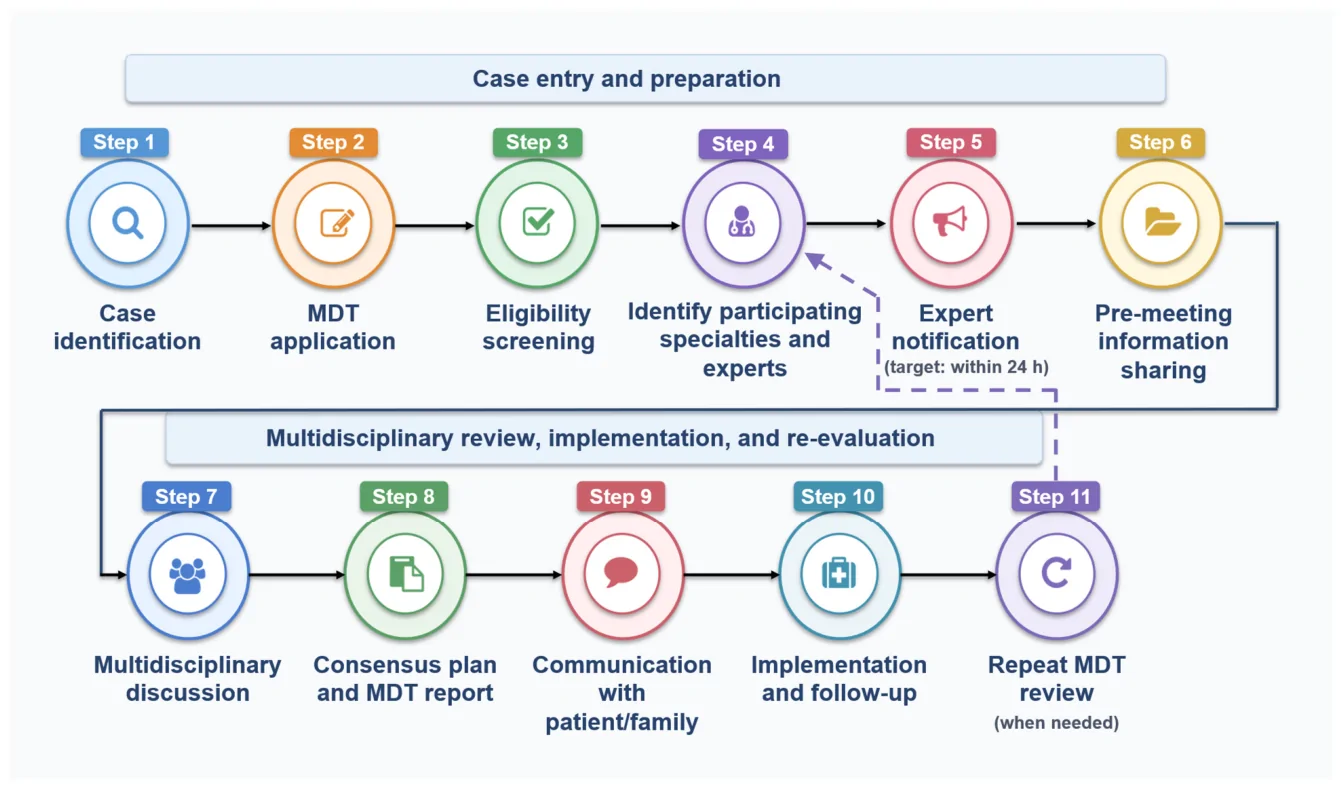
Standardized workflow of MDT care. The workflow comprises case entry and preparation followed by multidisciplinary review, implementation, follow-up, and re-evaluation, with repeat MDT review initiated when further multidisciplinary input is required. The figure illustrates the intended workflow rather than measured adherence to individual steps.

The hospital-wide MDT program is organized around a standardized workflow from case identification to follow-up and, when clinically indicated, repeat multidisciplinary review (Figure 3). Potential cases are identified according to institutional indications for MDT care, including difficult or critical illness, multisystem disease, rare disease, preoperative assessment for major surgery, comprehensive cancer treatment, and other complex conditions requiring cross-specialty coordination. Clinicians submit an MDT application through the MDT management platform, including relevant patient information, a clinical summary, preliminary diagnostic considerations, and the reason multidisciplinary input is required. Applications are then screened against the institutional eligibility criteria.

For eligible cases, the MDT center director identifies the participating specialties and experts required for the clinical problem and is expected to arrange their notification within 24 h of the application. Relevant medical records, laboratory findings, imaging studies, and other available clinical information are shared with participating experts before the meeting. During the MDT discussion, the attending physician presents the case and key clinical questions, participating experts provide discipline-specific assessments, and the group formulates a consensus management plan. The discussion is documented and an MDT report is generated.

The consensus plan is subsequently communicated to the patient and/or family by the attending physician and implemented by the responsible clinical team. Follow-up is incorporated into the workflow to assess subsequent clinical progress. When the clinical response is unsatisfactory, the clinical condition changes, or further multidisciplinary input is considered necessary, the case may undergo repeat MDT review. This workflow is intended to create a closed-loop pathway linking case selection, multidisciplinary decision-making, implementation, follow-up, and re-evaluation. Adherence to individual workflow steps was not systematically evaluated in this report.

### 3.4. Digital Information and Workflow Support

The hospital-wide MDT program is supported by the hospital information infrastructure and a dedicated MDT management platform. Integration with outpatient and inpatient electronic medical records, laboratory results, imaging studies, and other relevant clinical information enables participating clinicians to access case materials required for multidisciplinary review. The MDT management platform supports key workflow functions, including case application and management, expert scheduling and notification, pre-meeting information sharing, documentation of multidisciplinary discussions, and generation of consensus plans and MDT reports. These functions provide digital support for the standardized workflow described in Figure 3.

**Figure 4:**
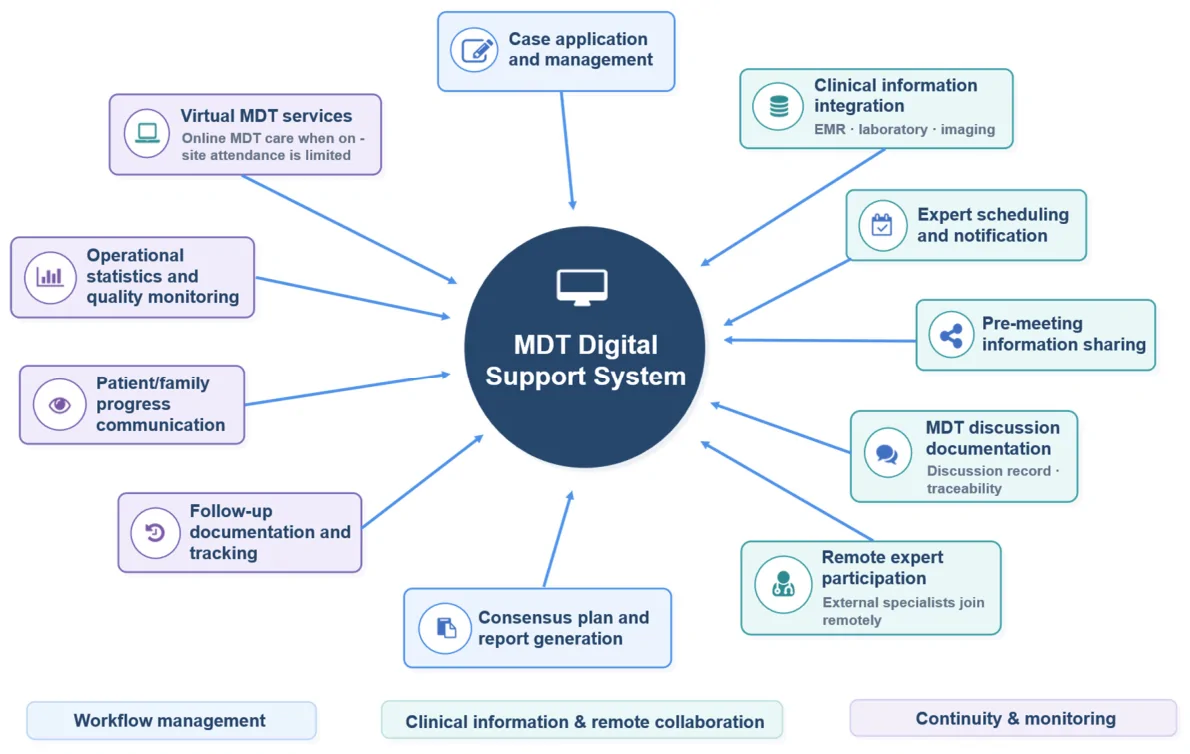
Digital functions supporting the hospital-wide MDT program. The figure summarizes the principal digital functions supporting workflow management, clinical information sharing and remote collaboration, and continuity and monitoring across the MDT process. Virtual MDT services refer to patient-level online MDT care, whereas remote expert participation refers to external specialists joining MDT activities remotely. EMR, electronic medical record.

The digital infrastructure also supports remote modes of MDT care. Patients who are unable to attend the hospital in person can receive virtual MDT services through the hospital’s online consultation system, while external specialists can participate remotely when additional expertise is required. The platform further supports longitudinal documentation of MDT activities, including follow-up information and access to relevant records across different stages of care. Patient-facing functions allow consultation progress to be communicated to patients and families, while aggregated operational data can be used for program monitoring and quality management. Figure 4 summarizes the principal digital functions supporting the hospital-wide MDT program.

### 3.5. Application Across Complex Clinical Domains

The hospital-wide MDT framework is applied across a range of complex clinical domains (Figure 2). The following examples illustrate how multidisciplinary expertise may be configured according to clinical needs and are not intended to represent individual case reports or outcome evaluations. In geriatric care, the MDT is led by geriatric medicine and may involve neurology, cardiology, endocrinology, nephrology, respiratory medicine, rehabilitation, nutrition, pharmacy, and other specialties according to individual patient needs. This multidisciplinary configuration reflects the domains typically considered in comprehensive geriatric assessment, in which medical, functional, psychological, and social problems are evaluated together to support an integrated care plan [11,12].

In cases involving gastrointestinal or microbiota-related problems together with urinary, nutritional, infectious, or medication-related issues, the Gastrointestinal and Microecology MDT Center can draw on expertise from urologic and renal services, nutrition, pharmacy, laboratory medicine, rehabilitation, and other relevant departments. Such cross-center and cross-departmental participation illustrates how the institutional MDT network can be configured around the needs of individual complex cases. This example is presented to illustrate multidisciplinary coordination rather than to infer that the MDT model itself improves specific clinical outcomes.

The same institutional framework is also applied in oncology, cardiovascular disease, neurologic disease, critical care, and other complex-care domains. Across these settings, the participating specialties and clinical priorities vary according to the case, while common requirements for coordination, documentation, implementation, and follow-up are maintained.

### 3.6. Operational Scale, Service Delivery, and Educational Activity

Annual operational indicators for the hospital-wide MDT program from 2021 to 2025 are summarized in Table 1. The recorded number of patients receiving MDT care increased from 32 in 2021 to 223 in 2025, with a modest decrease from 90 patients in 2023 to 87 in 2024. The number of formally established MDT centers increased from three in 2021 to seven in 2023 and remained stable thereafter. In parallel, the number of participating departments increased from four in 2021 to 23 in 2025, indicating broader cross-departmental participation within the hospital-wide MDT framework.

The mode of MDT service delivery also changed during the reporting period. Virtual MDT was the mode of first MDT contact for 27 of 32 patients (84.4%) in 2021 and 38 of 49 patients (77.6%) in 2022, during a period when COVID-19-related restrictions limited on-site attendance. From 2023 onward, on-site MDT became the predominant mode of first contact, accounting for 55 of 90 patients (61.1%) in 2023, 73 of 87 (83.9%) in 2024, and 170 of 223 (76.2%) in 2025. Virtual MDT nevertheless continued to be used as a complementary service modality when in-person attendance was restricted or impractical.

The educational component of the program was reflected in trainee participation in routine MDT activities. MDT sessions involving at least one trainee increased from 15 in 2021 to 119 in 2025. The number of unique trainees participating in MDT activities increased overall from 2 to 20 over the same period, with year-to-year variation. These indicators describe educational participation rather than educational effectiveness.

### 3.7. Implementation Challenges, Adaptive Responses, and Organizational Learning

Implementation of the hospital-wide MDT program required adaptation to challenges involving expert scheduling, cross-specialty disagreement, case selection, information completeness, implementation and follow-up, patient communication, sustained clinician participation, and cross-campus coordination (Table 2). Institutional responses combined standardized procedures, digital support, structured documentation, recognition of MDT-related workload and professional contributions, and repeat-review pathways.

The synthesized implementation experience highlighted several operational considerations identified during the development and operation of the MDT program, including clear governance, reliable administrative coordination, accessible clinical information, clinician participation, and closed-loop documentation and follow-up. Table 2 summarizes the challenges encountered, institutional responses, and practical implications drawn from this institutional experience. These practical implications represent context-dependent insights derived from the author team’s descriptive analysis of documented practices and implementation experience (rather than formal qualitative analysis). They may inform the development of similar initiatives in other institutional settings.

## 4. Discussion

This Project Report describes the evolution of a hospital-wide MDT model from specialty-based multidisciplinary practice into an integrated institutional coordination framework. Its distinctive contribution lies in documenting how MDT services across oncological and non-oncological domains can be brought together through a common organizational structure that links hospital-level governance, clinical-domain-based MDT centers, functional support, and shared operational procedures. Beyond describing the organizational structure itself, the report traces how case selection, expert coordination, clinical information preparation, multidisciplinary deliberation, documentation of consensus, patient communication, and follow-up are connected within a continuous care process. By presenting these organizational arrangements alongside multi-year operational indicators and implementation experiences, the report provides a practical account of how a hospital-wide MDT program has been organized and developed in a tertiary general hospital. This account may inform other hospitals seeking to establish or coordinate multiple specialty-based MDT services by offering organizational elements to consider and adapt to their own clinical needs, governance arrangements, staffing resources, and digital infrastructure. The expansion of MDT centers, participating departments, patient reach, and trainee involvement during 2021–2025 reflects increasing program scale and organizational reach. Nevertheless, these indicators provide a partial picture of program development, and a fuller assessment of changes in coordination, service quality, and implementation effectiveness would require additional evaluation. Taken together, the experience supports viewing MDT care not simply as a meeting of specialists but as a coordinated institutional process whose operation depends on the surrounding organizational infrastructure.

### 4.1. Governance and Standardization as Enabling Conditions

The interprofessional collaboration literature has identified organizational, team, and individual factors as important determinants of collaborative performance [4], while evidence that specific interprofessional interventions consistently improve clinical or service outcomes remains mixed and of low certainty [5]. In China, a systematic review identified substantial gaps in evidence linking teamwork structures and processes to organizational and patient outcomes [8], and qualitative research in county-level hospitals highlighted leadership, task design, and continuous improvement of team processes as important influences on team functioning [9]. The present model addresses several of these dimensions through designated clinical leadership, explicit case-selection procedures, standardized application and discussion processes, documented recommendations, and institutional mechanisms supporting participation.

Standardization is particularly relevant because MDT care requires substantial specialist time and coordination. Without appropriate case selection and structured preparation, multidisciplinary review may be delayed by incomplete information or used for cases that do not require cross-specialty input. Studies of oncology MDTs have similarly identified time pressure, incomplete information, attendance, leadership, workload, logistics, and team processes as factors influencing multidisciplinary decision-making [13,14]. In the present program, standardized applications, advance information sharing, designated clinical leadership, and documented consensus procedures are intended to support more focused and consistent multidisciplinary review. The implementation challenges summarized in Table 2 further indicate that governance and standardization require continuing operational adaptation rather than one-time institutional design.

### 4.2. Digital Infrastructure Should Support, Not Replace, Team Processes

Digital infrastructure represents an enabling layer of the hospital-wide MDT model. In a large tertiary hospital, identifying appropriate experts, assembling clinical information, documenting decisions, and coordinating subsequent care can generate substantial administrative and communication demands. Digital workflow functions can support these activities by linking case applications, clinical information, expert notification, documentation, and follow-up within a common workflow.

The experience during the reporting period also illustrates the complementary roles of virtual care and remote professional collaboration. Virtual MDT was used extensively during periods when on-site attendance was restricted, whereas on-site MDT became the predominant mode from 2023 onward while virtual services remained available as a complementary option. In parallel, remote participation enables external specialists to contribute when additional expertise is required. A scoping review of virtual and hybrid cancer MDT meetings similarly identified infrastructure, training, chairing, role clarity, and organizational logistics as important implementation factors [15]. These observations suggest that digitalization should be regarded as a means of supporting information access, coordination, and continuity rather than as a substitute for multidisciplinary clinical interaction.

### 4.3. Patient-Centeredness Requires Integration of Patient Preferences

Multidisciplinary consensus does not automatically constitute patient-centered care. Integrated-care frameworks emphasize coordination around individual needs, values, and preferences [2,3]. In the present model, the consensus plan is communicated to the patient and/or family by the responsible clinical team, allowing clinical recommendations to be explained and patient preferences to be considered before subsequent management is implemented.

This distinction is important because MDT decision-making may otherwise remain dominated by professional perspectives. Observational research in cancer MDT meetings has shown that individual patient characteristics and treatment preferences may receive limited attention during multidisciplinary deliberation [16]. Direct patient participation in MDT meetings is also not necessarily straightforward; multicenter observational evidence has demonstrated considerable variation in how such participation is organized and in the extent to which patients contribute to decision-making [17]. Future evaluation of the present model should therefore incorporate patient-reported experience, understanding of the management plan, participation in decision-making, and concordance between recommendations and patient preferences alongside conventional operational indicators.

The communication components described in this model can also be considered from a health communication and patient safety perspective. Previous studies have emphasized the importance of structured information exchange, standardized communication processes, and family-centered communication in reducing communication failures and supporting coordinated care [18,19]. In the present MDT model, professional communication is facilitated through pre-meeting preparation, multidisciplinary discussion, and documentation of consensus recommendations, while communication with patients and families is incorporated as part of the clinical workflow. However, these communication processes were not systematically evaluated in relation to adverse events, patient experience, or clinical outcomes. Future studies should examine whether structured communication within MDT models contributes to measurable improvements in patient safety and patient-centered outcomes.

### 4.4. Relevance to Complex Geriatric and Multisystem Care

The geriatric application illustrates why a hospital-wide MDT framework may be relevant beyond disease-specific settings such as oncology. Comprehensive geriatric assessment is inherently multidimensional and multidisciplinary, integrating medical, functional, psychological, and social domains in care planning [11,12]. Reviews of integrated care for older people likewise suggest potential benefits for selected service and care outcomes, although effects vary according to intervention design and context [20].

For patients with multimorbidity or multisystem problems, the practical role of MDT care may therefore extend beyond reaching a single diagnostic or therapeutic decision. Multidisciplinary review can provide a structure for reconciling competing treatment priorities, medication management, functional goals, rehabilitation, nutritional needs, organ-specific interventions, and subsequent follow-up. The gastrointestinal and microecology, urologic and renal, cardiovascular, neurologic, oncologic, and critical-care applications described in this report further illustrate how a common institutional framework can be adapted to different clinical domains while retaining shared expectations for coordination, documentation, and continuity.

### 4.5. Transferability to Other Tertiary Hospitals

The model may be adaptable to other tertiary hospitals because many of its core components are organizational rather than disease-specific: defined governance, accountable clinical leadership, explicit case-selection criteria, advance information preparation, structured consensus-building, documentation, follow-up, and digital support. A task-force report from a Korean tertiary hospital discussed digital infrastructure, multidisciplinary governance, and professional training as priorities for integrated community care [21]. A community-based gestational diabetes screening report illustrated how implementation procedures were adapted to local circumstances and stakeholder feedback [22]. The present report adds a context-specific account of how MDT services across oncological and non-oncological domains are brought together within a common hospital-level framework. This organizational perspective complements previous work; it does not establish that the individual components are novel or that this model is more effective than other MDT arrangements.

Transferability nevertheless requires contextual adaptation. Several organizational components described in this report, including defined governance responsibilities, clinical leadership, explicit case-selection criteria, structured information preparation, multidisciplinary consensus-building, documentation, and follow-up, may be adapted to other hospital settings. However, their implementation depends on local institutional conditions. Establishing hospital-level coordination and supporting functions requires appropriate organizational authority and administrative capacity, while the implementation of digital workflows depends on information-system maturity and available technical resources. Hospitals also differ in specialist availability, financing and payment arrangements, patient volume, and existing patterns of interdepartmental collaboration. Implementation science similarly emphasizes the influence of contextual determinants; the updated Consolidated Framework for Implementation Research organizes such determinants across the innovation, outer setting, inner setting, individuals, and implementation process [23]. A coordination structure that is feasible in a tertiary academic hospital may therefore require substantial modification in smaller or less-resourced institutions. The present model should consequently be interpreted as a set of potentially transferable operational components rather than as a fixed organizational blueprint.

### 4.6. Limitations and Priorities for Future Evaluation

This report is limited by its single-center, descriptive design and reliance on routine administrative and program records. Without a contemporaneous comparator or systematic patient-level outcome data, the findings cannot establish whether MDT care improves clinical or health-service outcomes. The available operational data may also be affected by limitations in completeness and measurement, and the report does not quantify how often MDT review changes diagnosis, treatment, or subsequent management. A consistently defined denominator of patients eligible for MDT consultation was not available; therefore, MDT coverage or penetration rates could not be calculated. Patient characteristics, case complexity, participating specialties, implementation of recommendations, and follow-up outcomes were not systematically compared between virtual and on-site MDT care. In addition, the implementation lessons were synthesized from institutional documentation and experience rather than formal qualitative research, and their transferability may depend on local organizational structures, specialist resources, and digital capabilities.

Future evaluation should use a prospectively defined framework combining process, patient-centered, clinical, and economic measures. Relevant indicators may include timeliness of MDT review, information completeness, implementation of recommendations, patient experience and participation, selected clinical and health-service outcomes, staff workload, resource use, and cost-effectiveness. Such evaluation would help determine which components of the model provide measurable value and under what organizational conditions they are most sustainable.

## 5. Conclusions

The experience of The Second Affiliated Hospital of Nanjing Medical University suggests that hospital-wide MDT care is best understood as an institutional coordination framework rather than a series of isolated specialist meetings. Its implementation appears to depend on the integration of governance, standardized workflows, information sharing, documented multidisciplinary decision-making, longitudinal follow-up, digital support, and sustained clinician participation.

This Project Report does not establish comparative clinical effectiveness, but it provides practical implementation insights that may inform similar initiatives in tertiary general hospitals. Prospective evaluation is needed to determine the effectiveness, sustainability, and transferability of the model.

## Figures and Tables

**Table 1:** Annual operational indicators of the hospital-wide MDT program, 2021–2025.

Year	MDT Patients (*n*)	On-Site MDT (*n*)	Virtual MDT (*n*)	MDT Centers (*n*)	Departments (*n*)	MDT Sessions with Trainees (*n*)	Trainees (*n*)
2021	32	5	27	3	4	15	2
2022	49	11	38	5	7	22	10
2023	90	55	35	7	10	41	13
2024	87	73	14	7	15	56	8
2025	223	170	53	7	23	119	20

Note: Patients and trainees were counted as unique individuals within each calendar year. On-site and virtual MDT were classified according to the mode of the patient’s first MDT encounter in that year. Detailed operational definitions are provided in Section 2.3. These indicators represent recorded MDT activity and do not estimate population-level coverage or referral penetration.

**Table 2:** Major implementation challenges, institutional responses, and practical implications derived from MDT implementation experience.

Implementation Challenge	Institutional Response	Practical Implication
**Expert scheduling and participation**	Use recurring meeting times where feasible; arrange expert participation in advance; and establish backup arrangements.	Embed scheduling and contingency arrangements in routine coordination.
**Cross-specialty disagreement**	Use a structured discussion sequence led by the MDT center director; obtain discipline-specific input; document areas of disagreement and the final consensus; and seek additional expert input when necessary.	Disagreement should be managed through explicit consensus procedures and transparent documentation rather than informal resolution.
**Case selection and application quality**	Apply explicit institutional eligibility criteria and standardized application templates with required clinical information, and screen applications before organizing the MDT review.	Clear entry criteria and structured applications help focus MDT resources on cases requiring multidisciplinary input.
**Pre-meeting information completeness and interoperability**	Integrate electronic medical records, imaging, and laboratory data; supplement unavailable external records when needed; review information completeness before the meeting; and provide technical support for platform use.	Complete and accessible clinical information was an important operational consideration in multidisciplinary review, while digital integration required ongoing operational support.
**Implementation, follow-up, and repeat review**	Archive the MDT report in the medical record; document implementation of the consensus plan and reasons for clinically necessary deviations; maintain follow-up; and initiate repeat MDT review when the clinical condition changes or further multidisciplinary input is required.	Closed-loop documentation and follow-up are needed to connect consensus decisions with subsequent care and re-evaluation.
**Patient/family communication and treatment preferences**	Explain the purpose and process of MDT care, communicate the consensus plan in understandable language after multidisciplinary review, and document major concerns, preferences, and reasons for non-acceptance when relevant.	Patient communication should support informed choice and consideration of patient preferences in MDT decision-making.
**Sustained clinician participation and institutional recognition**	Recognize MDT participation as part of routine workload and professional contribution through institutional support mechanisms, with acknowledgement of substantive clinical contributions.	Recognize MDT work as part of institutional workload and professional contribution.
**Cross-campus and remote coordination**	Use hospital-level governance, shared operational standards, coordinated case-transfer pathways, and remote participation when additional expertise or cross-campus support is required.	Common standards and centralized coordination are important for consistency across campuses and service locations.

Note: The table summarizes implementation challenges and institutional responses documented within this hospital-wide MDT program. The practical implications represent context-dependent insights derived from the author team’s descriptive analysis of documented practices and implementation experience, rather than formal qualitative analysis or empirically established determinants of MDT implementation success.

## Data Availability

The aggregated data supporting the findings of this report are presented in the article. The underlying institutional records are not publicly available because of institutional data-governance considerations but may be available from the corresponding authors upon reasonable request and subject to institutional approval.
